# Advancing Toward HIV-1 Vaccine Efficacy through the Intersections of Immune Correlates

**DOI:** 10.3390/vaccines2010015

**Published:** 2013-12-27

**Authors:** Georgia D. Tomaras, Barton F. Haynes

**Affiliations:** 1 Departments of Surgery, Immunology, and Molecular Genetics and Microbiology, Duke Human Vaccine Institute, Durham, NC 27710, USA; 2 Departments of Medicine and Immunology, Duke Human Vaccine Institute, Durham, NC 27710, USA

**Keywords:** HIV-1, vaccine, immune correlate, protection, immunity, clinical trials

## Abstract

Interrogating immune correlates of infection risk for efficacious and non-efficacious HIV-1 vaccine clinical trials have provided hypotheses regarding the mechanisms of induction of protective immunity to HIV-1. To date, there have been six HIV-1 vaccine efficacy trials (VAX003, Vaxgen, Inc., San Francisco, CA, USA), VAX004 (Vaxgen, Inc.), HIV-1 Vaccine Trials Network (HVTN) 502 (Step), HVTN 503 (Phambili), RV144 (sponsored by the U.S. Military HIV Research Program, MHRP) and HVTN 505). Cellular, humoral, host genetic and virus sieve analyses of these human clinical trials each can provide information that may point to potentially protective mechanisms for vaccine-induced immunity. Critical to staying on the path toward development of an efficacious vaccine is utilizing information from previous human and non-human primate studies in concert with new discoveries of basic HIV-1 host-virus interactions. One way that past discoveries from correlate analyses can lead to novel inventions or new pathways toward vaccine efficacy is to examine the intersections where different components of the correlate analyses overlap (e.g., virus sieve analysis combined with humoral correlates) that can point to mechanistic hypotheses. Additionally, differences in durability among vaccine-induced T- and B-cell responses indicate that time post-vaccination is an important variable. Thus, understanding the nature of protective responses, the degree to which such responses have, or have not, as yet, been induced by previous vaccine trials and the design of strategies to induce durable T- and B-cell responses are critical to the development of a protective HIV-1 vaccine.

## 1. Introduction

### Insights from Multiple Measurements: Immune Responses, Virus Sieve Analysis and Host Genetics

**Figure 1:**
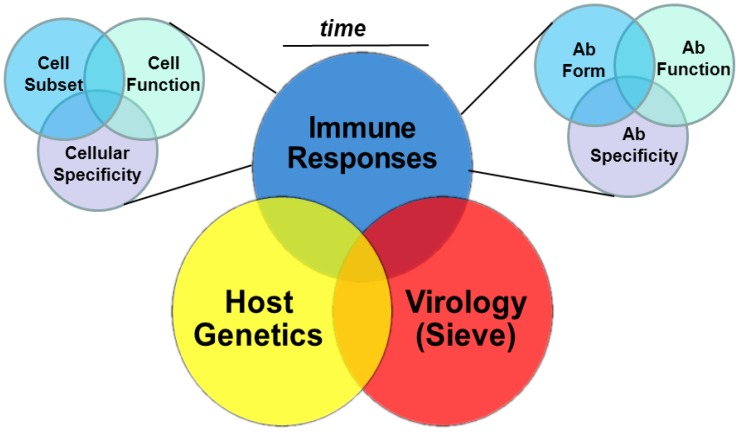
Overlapping regions among distinct analyses are targets for novel insights. The intersection of correlate analyses (shown as overlap of circles) involving immune responses (innate, cellular, humoral), host genetics (e.g., FcR expression, HLA), and HIV-1 virus sieve analyses are areas of the highest information content for follow-up studies. A subset of independent immune response measurements (e.g., cellular and humoral responses top left and right) can also overlap to point to similar potential mechanism(s) of protective immunity. (Ab Form: antibody subclass, isotype, dimeric, *etc*.) The magnitude and quality of vaccine induced immune responses differ in time (black line) post vaccination and may influence vaccine efficacy.

The path forward to an efficacious HIV-1 vaccine will be expedited via the analysis of immune correlates from past and future HIV-1 vaccine efficacy trials. Approaches that aim either to improve upon current findings from a partially efficacious vaccine strategy or approaches toward the generation of broadly neutralizing antibodies by rational immunogen design strategies are likely to be fast-tracked through careful probing of the intersections of immune correlate analyses within and among human vaccine clinical trials. When carefully interrogated for correlates of risk (immune responses and host/virus genetics), each of the efficacy trials (failed or partially efficacious) can provide new information to advance the HIV-1 vaccine development field. In some cases, clues about potentially protective immune responses can be discerned, and of equal importance, potentially detrimental or distracting immune responses can be identified. Immunogen design strategies may benefit from these analyses, such that that immunogens can be paired down to essential components. Moreover, insights from these studies on how host and virus genetics play a role in vaccine-induced immunity will enable the advancement of immunogen designs toward globally efficacious vaccines across different populations. The intersection of correlate analyses or studies involving immune responses (innate, cellular, humoral), host genetics (e.g., FcR expression, HLA) and HIV-1 virus sieve analyses (virus sequence analysis of all infections (placebo and vaccine groups) to determine if there were specific genetic attributes of infecting viruses that were significantly different between the two groups) are areas of the highest information content for follow-up studies (Figure 1). In this review, the correlate analyses for HIV-1 vaccine efficacy trials are described, with an emphasis on those studies that examine the intersection of different components and analyses.

## 2. HIV-1 Vaccine Efficacy Trials

Due to the uniqueness of each efficacy trial and outcome, each study can provide information regarding the minimum bar to overcome to achieve HIV-1 vaccine efficacy. Even when there is no overall vaccine efficacy, the heterogeneity of immune responses among vaccines has enabled follow-up studies to identify associations of immune responses with HIV-1 infection risk in subsets of vaccines. Furthermore, an integral part of understanding immune correlates of protection is to also define patterns of immune responses that associate with non-protective vaccines. One of the major goals for the HIV-1 vaccine field is to define correlates of protection from HIV-1 infection, such that the field has biomarkers that will clearly predict vaccine outcome in HIV-1 vaccine efficacy trials.

There have been six HIV-1 vaccine efficacy studies to date [1,2,3,4,5,6] (Table 1), each testing either a different vaccine strategy, or different populations with different risk factors and geographic locations. HIV-1 vaccine efficacy studies have been analyzed for correlates of infection risk; however, a correlate of protection for an HIV-1 vaccine has not yet been identified. Both the non-efficacious vaccines [7,8,9,10,11] and the one partially efficacious HIV-1 vaccine [12,13,14,15] trial have yielded several correlates of infection risk; although more weight is given to those findings from trials where there was overall vaccine efficacy. In total, three of these studies (VAX004, Step, and RV144) found significant correlations with HIV-1 infection risk/incidence and two of these studies (Step and RV144) identified potential sites of immune pressure on the virus (virus sieve).

The terminology employed for describing correlates and surrogates for protection from HIV-1 infection have been the focus of ongoing discussion and clarification [16,17,18,19]. It is generally agreed upon that for any one of the correlates of infection risk identified in RV144 to be considered a correlate of protection that reliably predicts protection from HIV-1 infection, it must be tested and proven in another clinical trial either with the same setting or in a different vaccine setting [18]. Ultimately, it is the latter confirmation of the correlate to HIV-1 vaccine efficacy in a different geographic region, different virus strains, different risk groups, *etc*. that is the most sought after for the development of a global HIV-1 vaccine.

**Table 1:** HIV-1 vaccine efficacy trials and immune correlates.

HIV-1 Efficacy Trial	Vaccine Strategy	Risk Population	Geographic Location	Vaccine Efficacy Outcome ^a^	Correlates of HIV-1 Risk/Incidence	Evidence of Immune Pressure (Virus Sieve)
**1. VAX003 [2]** **(Phase III)**	Protein/Alum (CRF01_AE/Clade B Env)	Injection Drug Users (IDU)	Thailand	No Efficacy	No [2]	No [20]
**2. VAX004 [1]** **(Phase III)**	Protein/Alum (Clade B Envs)	MSM ^b^/High Risk Women	USA	No Efficacy	Yes [7,8,9]	No [21,22]
**3. Step HVTN502 [3] (Phase IIb)**	Ad5 Vector (Clade B Gag/Pol Nef)	MSM/ High Risk Heterosexual Men and Women	North and South America, Australia, Caribbean	No Efficacy (Efficacy futility determined at first interim analysis after full enrollment)	Yes [10,11]	Yes [23]
**4. Phambili** **HVTN503 [4] (Phase IIb)**	Ad5 Vector (Clade B Gag/Pol/Nef)	Heterosexual men and women	South Africa	No Efficacy (Vaccinations discontinued: early unblinding due to Step results)	*None reported*	*None reported*
**5. RV144 [5]** **(Phase III)**	ALVAC vector (Clade B Gag/Pro + CRF01_A/E Env) + Protein/Alum (CRF01_AE/Clade B Env)	Community ^c^	Thailand	31.2% Efficacy	Yes [12,13,14,15]	Yes [24,25]
**6.HVTN505 [6] (Phase IIb)**	DNA + Ad5 Vector (Clade A,B,C *Env*, *Clade B Gag/Pol*)	MSM and TG ^d^ (Ad5 seronegative, Circumcised)	USA	No Efficacy (Efficacy futility determined at first interim analysis after full enrollment )	*Studies* *In Progress*	*Studies* *In Progress*

Vaccine-induced immune responses have been studied to identify immune correlations with infection risk and evidence of virus sieve that can inform the design and evaluation of the next phase of vaccine efficacy trials (shaded boxes indicate reported correlations). Immunizations in two efficacy studies (Phambili/HVTN 503, HVTN 505) were stopped; however, follow-up of participants continues. ^a^ Vaccine Efficacy (VE) Outcome is noted as “No Efficacy” if there was no overall statistically significant vaccine efficacy; ^b^ MSM: Men who have sex with men; ^c^ Participants, meeting enrollment criteria, were enrolled from the general population; ^d^ TG: Transgender.

## 3. Cellular Immunity and HIV-1 Vaccine Efficacy Trials

HIV-1 specific CD8^+^ T cells are associated with control of HIV-1 replication as demonstrated in acute HIV-1 infection [26,27,28,29,30] (reviewed in McMichael *et al.* [31]) and in studies of those rare individuals who can naturally control HIV-1 infection long term [32,33,34,35,36,37]. Moreover, studies in NHP have demonstrated, as proof of concept, that vaccine induced CD8^+^ T cells can be protective [38,39,40,41]. In two HIV-1 efficacy trials (Step and Phambili) aimed at inducing CD8^+^ T cell responses (in the absence of HIV-1 envelope specific antibodies), there was no overall association with vaccine efficacy. However, these studies indicated that there were vaccine-elicited CD8^+^ T cells that could impact virus replication; although not sufficient enough to provide overall decreased risk of infection in the trial (either due to low magnitude/breadth/function of T cell responses and/or pre-existing vector immunity *etc.*). HVTN 505, also designed to target the induction of CD8^+^ T cell responses (as well as HIV-1 envelope specific antibodies), was discontinued and unblinded due to lack of efficacy. Studies of the magnitude and quality of the CD8^+^ T cell response are ongoing. Since there was evidence of increased infections in two of the Ad5 vector based vaccine studies, Step and Phambili [42] but not in HVTN 505 [6], there is a greater emphasis on understanding immune responses to vaccine vectors and impact on subsequent immunity. Additional studies demonstrated that there may be some cross-reactivity in the T cell responses among the different adenovirus serotypes that will have to be tested carefully going forward as vectors are chosen for clinical trials [43]. An alternate interpretation is that perhaps many other types of HIV-1 vaccines induce some level of CD4^+^ T cell activation; however, the key is to have sufficient overall HIV-1 specific immunity to tip the balance toward protection. Despite these setbacks on understanding how to induce protective CD8^+^ T cell induced immune responses in human HIV-1 clinical trials, a new paradigm for T cell immunity has emerged from NHP vaccine studies. Picker and colleagues report on a CMV based vaccine regimen that protects NHPs from infection and induces a novel subset of CD8^+^ T cells that can broadly recognize HIV-1 epitopes through class II restriction [38]. The series of papers by Picker and colleagues provide new hypotheses to test for inducing effective cellular immunity, both in terms of both new immunogen vector design and new subsets of CD8^+^ T cells to target with vaccine approaches. 

### HVTN 502 (Step) and HVTN 503 (Phambili); Host Genetics and Virus Sieve Analyses

The Step study [3,44] as well as the Phambili study [4] were test of concept studies (double-blind, randomized, placebo-controlled) for induction of HIV-1 specific T cells responses. These trials tested the concept of a replication defective adenovirus serotype 5 (Ad5) vector with clade B HIV-1 genes (*gag*, *pol nef*) (MRKAd5) to decrease virus load in those who became infected. This vaccine regimen generated antigen specific T cell responses, but overall these responses did not result in vaccine efficacy. An increased rate of HIV-1 infection was observed in vaccines compared to placebos that was associated with being uncircumcised and having pre-existing Ad5 antibodies (reviewed in Gray *et al.* [45]). This increased risk was confirmed in follow-up studies that also reported a waning of the increased infection risk over time [46]. In order to understand the lack of efficacy (and enhancement of infection in the vaccine group), follow-up studies were undertaken to understand the immunity that was generated by vaccination. One of the goals of these follow-up studies was to examine potentially protective responses that may be subdominant in the population but may impact either acquisition or disease progression in some of the vaccines. Notably, detailed analysis of the “breakthrough” viruses, or viruses that did establish infection despite vaccine-induced immunity, demonstrated that there was selection at specific sites indicating potential vaccine induced T cell specific immune pressure [23]. Notably, the antigen specific CD8^+^ T cells responses induced by Ad5 vaccination were to HIV-1 epitope hotspots that were different than those generated in natural infection and were also directed to highly variable regions that likely can tolerate escape mutations from vaccine induced immune responses [47]. Host genetics also played a role in immunity, since those vaccines with HLA alleles (B*27, B*57, B*58:01), known to be associated with HIV-1 control, had lower viral load [48] and their CD8^+^ T cells exhibited greater killing in *in vitro* assays [49]. Recent analysis of those infected within a year of their last vaccination, identified that total T cell breadth and total magnitude of the immune response after the immunization series and prior to infection significantly correlated with lower mean viral load [50]. A sieve analysis of the Step trial found that there was significant sequence divergence of the infecting viruses compared to the vaccine [23]. However, despite an observed anamnestic T cell response (from vaccine-induced immunity) after infection, the specific T cell responses measured as part of the evaluation of the trial did not correspond with the sieve findings [51]. Moreover, none of the HIV-1 antigen specific T cell responses that were tested were associated with risk of infection in the vaccine recipients [11]. Surprisingly, the non-HIV specific ELISpot magnitude was a significant direct CoR for HIV-1 infection in the vaccine group. Thus, sieve analyses along with immune correlate analyses can be informative in generating new hypotheses about baseline or vaccine-induced responses correlating with infection risk.

## 4. Humoral Immunity and HIV-1 Vaccine Efficacy Trials

Four of the HIV-1 vaccine efficacy trials induce HIV-1 Env-specific antibody responses by three diverse strategies: recombinant HIV-1 gp120 immunogen alone (VAX003 and VAX004); vector prime with gp120 boost (RV144); and DNA prime, vector boost (HVTN 505). Although VAX003 and VAX004 efficacy trials each tested gp120 protein only as an immunogen strategy to induce humoral responses, these trials were distinct in that they were conducted in different risk populations (injection drug users (IDU) *vs*. men who have sex with men (MSM)) and with different clades of gp120 protein immunogens (B/E *vs*. B/B). Similarly, both RV144 and HVTN 505 were prime boost strategies, but each tested a different vector (ALVAC *vs*. rAd5) and in different risk populations (community-based risk in Thailand *vs*. MSM in the U.S.). It is unknown whether the same mechanisms of protection against HIV-1 acquisition will be similar or quite distinct among the different risk populations/transmission routes. Thus, there are four major differences across these HIV-1 vaccine efficacy trials that need to be considered when comparing studies: (1) differences in the HIV-1 gp120 sequence content; (2) differences in the approach (prime/boost and vector *vs*. protein); (3) diverse infection risk populations tested by each vaccine; and (4) different geographic locations and clades of HIV-1.

### 4.1. VAX003 and VAX004: Immune Response and Host Genetics

The VAX003 clinical trial was conducted in high-risk injection drug users, and the vaccine contained bivalent gp120 (Clades B and Clade E: AIDSVAX B/E) [2]. As part of the primary analysis of vaccine efficacy for VAX003, pre-specified antibody variables (V2 and V3 binding antibodies to a vaccine strain envelope (A244), gp120 binding antibodies (A244 and MN), CD4 blocking antibodies and neutralizing antibodies to HIV-1 MN) were found not to be correlates of risk. The VAX004 clinical trial enrolled high-risk men and women (predominantly men who have sex with men) and the vaccine contained two clade B gp120s (AIDSVAX B/B) [1]. Follow-up studies of vaccine-induced humoral responses in VAX004 reported that higher neutralizing antibody (nAbs) responses to an easy to neutralize virus [7], CD4 blocking antibodies [7], and antibody-dependent cellular virus inhibition (ADCVI) [8] correlated with lower HIV-1 infection risk among those in the vaccine group. Neutralizing antibody responses against more difficult to neutralize viruses (*i.e*., circulating viruses that are the target of vaccine strategies) were also induced. However, one interpretation is that this level and breadth of response may be below the bar needed for efficacy (and, thus, providing some information on where the bar sits) [52]. Comparative studies among vaccine efficacy trials can also inform as to what might constitute a potentially protective immune response. Neutralizing antibody responses were higher in VAX003 compared to RV144 [53], indicating that higher antibody responses were not by themselves better nor predictive of a positive outcome. Insights from analyses of host genetics suggest that although there was no overall increased infection in the VAX004 vaccine trial, the Fcγ receptor IIIa genotype (VV genotype, encoded by an allele with a valine (V) a position 158) was associated with an increased rate of HIV-1 infection in low risk vaccines, but not in high risk vaccines [9].

### 4.2. RV144

RV144 is the only HIV-1 vaccine efficacy trial in which the vaccine showed decreased transmission *versus* the placebo group, with an estimated vaccine efficacy of 31% [5]. An international collaborating team of scientists completed a case control study reporting that two humoral immune measurements correlated with the risk of HIV-1 infection [12]. IgG antibodies to the V1/V2 region of HIV-1 gp120 correlated with a decreased risk of infection [12,13,14,15], and a plasma HIV-1 envelope-specific IgA score correlated with increased risk of infection in the vaccine arm (decreased vaccine efficacy) [12,54]. Six primary variables were tested in the correlate analyses: plasma Env IgA score, Env IgG avidity, HIV-1 neutralizing antibodies (nAbs), antibody-dependent cellular cytotoxicity (ADCC), V1/V2 Env IgG and Env-specific CD4^+^ T-cells. Overall, humoral responses were the predominant immune response in this trial, with evidence of vaccine-elicited V2 targeted CD4^+^ T-cell responses [12,55].

#### 4.2.1. Antigenicity of Immunogens

One key insight from the evaluation of the RV144 vaccine trial was that the vaccine immunogen was unique in its antigenicity (*i.e.*, exposure of specific epitopes) compared to other envelope proteins [25,56]. Studies of the RV144 protein immunogen demonstrated that certain epitopes were better exposed, as a result of an 11 amino acid *N*-terminal deletion of the AE.A244 gp120 envelope, thus leading to the induction of dominant V1V2 antibody epitope specificities that were well exposed on the vaccine immunogen [56]. In particular, the A244 gp120 used in RV144 was antigenic for both linear V2 epitopes bound by strain-specific V2 antibodies, as well as for conformational V1V2-glycan epitopes bound by V1V2 broad neutralizing antibodies (BnAbs) [25,56]. Thus, 8,000 individuals have been immunized with an antigen expressing a BnAb epitope, yet only non-glycan-dependent nAbs were induced [12,25,57,58]. Thus, in addition to defining correlates of protection in RV144, a second critical reason to study vaccine responses in RV144 is to understand why BnAbs were not induced by an antigenic immunogen. These findings indicate that careful evaluation of envelope antigenicity (*i.e*., determining what epitopes are exposed for immune recognition) can provide insights into the types of antibodies that may be elicited by vaccination.

#### 4.2.2. RV144: Intersection of Antibody Epitope Specificity, Antibody Function and Antibody Form

Further analyses of the six primary variables in the RV144 correlate analyses using interaction models was one way of examining the role of multiple immune measurements in assessing correlates of HIV-1 infection risk. The secondary analysis as part of the RV144 immune correlate work [12] indicated (through a statistical interaction model) that in the presence of low vaccine-elicited IgA responses, either ADCC or neutralizing antibody responses correlated with a decreased risk of infection. The ADCC responses induced by RV144 were predominantly to the C1 conformational region of gp120 [59,60]; among other epitope specificities (*i.e*., V2, V3) [25]. Thus, one hypothesis from the RV144 correlate study was that C1 region Env-specific IgA could block C1-specific ADCC by binding to the same epitopes on infected cells, but without the functional engagement of NK cells that is needed to mediate ADCC. Indeed, RV144-induced Env IgA was shown to block C1 region-specific IgG-mediated antibody-dependent cellular cytotoxicity by natural killer cells [54]. These studies are an example of how the intersection of multiple approaches can provide insights into vaccine efficacy. Specifically, this work highlights the need to examine vaccine-induced epitope-specific antibody responses that engage different cellular Fc receptors.

#### 4.2.3. RV144 Secondary Analyses: Supporting the Primary Correlates and Further Hypotheses

Together, the two immune correlates of HIV-1 infection risk in RV144, along with data from the secondary analyses and follow-up studies, indicate that evaluating antibodies that target circulating strains in the population being tested as well as cross-clade epitopes may play a role in understanding vaccine efficacy. Circulating Env IgA responses to the C1 region in gp120 of a circulating strain in Thailand (CRF01_AE) was the strongest statistical correlate of HIV-1 infection risk in RV144. Moreover, IgG responses to the V2 region of CRF01_AE Env significantly correlated with a decreased risk of infection among the different clades of V2 responses tested in the peptide microarray assay [12,15]. Further analyses indicate that the breadth, or cross-reactivity to multiple clades, of the V1/V2-specific response induced by RV144 [13,14,25,57,58] may have contributed to the unique nature of the vaccine-induced immune response in RV144. Further studies in other vaccine trials will be able to test these specific hypotheses to see if vaccine-induced immunity that can broadly target circulating strains will be critical for vaccine-mediated protection from HIV-1 infection.

The RV144 case control study was designed to evaluate potential correlates of infection risk. Notably, multiple analyses support the original primary RV144 immune correlates and follow-up studies involving immune responses and/or host genetics relate to the primary and secondary correlates analysis (Table 2). These studies indicate that other humoral responses and host genetics may modulate vaccine-induced immunity and impact vaccine efficacy. Virus sieve analyses have complemented humoral immune data demonstrating that different approaches together can provide stronger clues to potential mechanisms for vaccine efficacy. Although there were only six primary immune measurements tested in the case control analysis, other measurements as part of the RV144 case control study [12] and as follow-up analyses [13,14,15,54] were significantly associated with HIV-1 infection risk (statistical results with *p* < 0.05) (Table 3). Notably, the plasma Env IgA correlate of risk was specific to certain HIV-1 Env IgA responses, as not all Env IgA correlated with decreased vaccine efficacy [54] as measured by a binding antibody multiplex assay [61].

#### 4.2.4. RV144 Studies: Virus Sieve Analysis and B-Cell Repertoire

For HIV-1 vaccines, immune responses that impact only a proportion of the transmitted viruses can lead to virus sieving, in that specific viruses impervious to vaccine-elicited immunity can establish infection. These virus sieve analyses in concert with probing vaccine-induced immune response can lead to hypotheses of specific immune responses that may prevent the acquisition of some viruses. Moreover, analyses of viruses escaping from vaccine-induced immunity can provide a roadmap for critical targets on infectious virions that need to be targeted by an HIV-1 vaccine to substantially increase vaccine efficacy. Ongoing analyses of the changing complexity of circulating viruses may be critical for understanding the virus target in specific populations. 

Additional independent studies examining breakthrough viruses in RV144 vaccines (sieve analyses) and the generation of monoclonal antibodies from the B-cells of vaccine recipients have pointed toward a specific site within the V2 region of the HIV-1 envelope. V2 mAbs derived from RV144 vaccines bound to an epitope centered on K169 of the V2 region [25], and a sieve analysis [24] focused on this region found K169 to be a critical site of immune pressure. Thus, if the infecting strain of HIV-1 matched the RV144 vaccine AE.V2 region at K169, the vaccine efficacy was 48%. Thus, we have proposed that one way to enhance the efficacy of follow-up trials to RV144 would be to include additional Envs or V2 peptides with V2 sequences not covered in the original RV144 envelope sequences. Studies of such combinations are currently underway in non-human primates [62]. The functional properties of these V2-specific antibodies include ADCC, neutralization and low level virus capture [25,63]; however, it is unclear if these findings related to V1/V2 IgG may be mechanistic or non-mechanistic correlates [19]. Thus, the combination of the initial analysis of the V2-directed humoral responses induced by RV144, work identifying that V1V2 (K169) IgG3 correlated with a decreased risk of infection [14], the mAb work that identified the importance of a lysine in position 169 of the V2 loop and virology work that resulted in the demonstration of K169 as a site of immune pressure, together, reinforced the hypothesis that the induction of V2-specific antibodies is important to test as a correlate of protection in further HIV-1 vaccine efficacy trials. 

**Table 2:** RV144 correlate analyses: intersections with virology and host genetics.

Type of Analysis	Description of Outcome	Overlap or Intersections with Other Measurements
Follow-up studies to the RV144 immune correlate analysis both support and extend the original report by additional work involving the generation of monoclonal antibodies (mABs), virology (virus sieve analysis) and host genetics.		
V2 Virus Sieve Analysis [24]	Vaccine efficacy was higher against viruses matching the vaccine at position 169 in Env (another signature at position 181 that mismatched the vaccine was also identified as unrelated to the V2 site).	To date, five follow-up studies support the identification of V1V2 IgG correlate [12], and the virus sieve analysis in the V2 loop are consistent with these findings. (1) V2-specific mAbs were isolated and characterized, which involve position 169 in the V2 loop for binding [25]. (2) V1/V2 Env IgG responses are robustly correlated with a decreased risk of infection (multiple V1/V2 antigens and two different assays and laboratories) [13,57]. (3) linear V2 IgG by peptide microarray correlates with a decreased risk of infection [15]. (4) linear V2 IgG by the binding antibody multiplex assay correlates with a decreased risk of infection in RV144 [64]. (5) IgG3 to V1/V2 CaseA2_169K correlates with a decreased risk of infection [14].
V3 Virus Sieve Analysis [65]	Vaccine efficacy was higher against viruses matching the vaccine in the Env V3 loop.	V3 IgG correlates with a decreased risk of infection [15].
Host Genetics: Human Leukocyte Antigen (HLA) Class I [66]	There was an association of the HLA Class I allele (A* 02) with HIV-1 infection risk and the two identified antibody correlates in RV144 vaccines.	This analysis of host genetics for HLA Class I alleles intersects with both the V1/V2 IgG response and the Env IgA response. (1) Vaccine efficacy for viruses with a lysine at position 169 was higher in those with the HLA A* 02 allele; (2) there was a direct correlation between plasma C1 Env IgA response and infection rate in the A* 02 (−) subgroup, but not the A* 02 (+) subgroup.
Host Genetics/HLA Class II [67]	There was an association of the HLA Class II alleles with HIV-1 infection risk and the plasma Env IgA correlate in RV144.	HLA II allele, DQB1* 06, had a significant interaction with the plasma Env IgA responses, such that DQB1* 06 had a significant effect on HIV-1 infection among the high IgA responders.
Host Genetics/FcγRIIC [68]	There was an association of FcγRIIC with vaccine efficacy.	This analysis of host genetics for HLA Class I alleles intersects with both V2 sieve analysis and the Env IgA response. (1) Vaccine efficacy for viruses with a lysine at position 169 was higher in those with at least one FcγRIIC-118l allele; (2) direct correlation of plasma Env IgA with infection risk was only when FcγRIIC-118l was present.

**Table 3:** RV144 secondary and follow-up immune measurements: associations and interactions.

RV144 Immune Measurement	Description of Outcome	Overlap or Intersections with other Measurements
**Interaction Models of Primary Correlate Analysis** The six primary immune measurements evaluated in the RV144 correlate analysis were chosen in part based on each one representing distinct immunological space. Two of these measurements (V1/V2 IgG and Env IgA) significantly correlated with the risk of HIV-1 infection as reported in Haynes *et al* [12]. Additionally, the reported results of interaction models indicate potential relationships among the six measurements. Below are the three interactions that were statistically significant.		
1. Interaction of Plasma Env IgA and IgG Env Avidity [12]	In the presence of low plasma Env IgA, avidity to vaccine strain Env correlated with a decreased risk of infection.	HIV-1-Specific Plasma IgA Env Breadth Score. IgG Env avidity.
2. Interaction of Plasma Env IgA and antibody-dependent cellular cytotoxicity (ADCC) [12,54,60]	In the presence of low plasma Env IgA, ADCC correlated with a decreased risk of infection.	HIV-1-Specific Plasma IgA Env Breadth Score antibody-dependent cellular cytotoxicity (ADCC). Follow-up mechanistic studies demonstrated that conformational C1-specific IgA antibody can block IgG-mediated ADCC (NK effectors) [54] .
3. Interaction of Plasma Env IgA and NAb [12]	In the presence of low plasma Env IgA, Nab correlated with a decreased risk of infection.	HIV-1-Specific Plasma IgA Env Breadth Score. Neutralizing Antibody (NAb) Score.
**Secondary Immune Measurements** A series of immune measurements, in addition to the preselected primary six variables, were evaluated for correlations with the risk of HIV-1 infection. Of all the secondary immune variables, eight of these had a *p*-value of < 0.05. Three of these are related to the direct correlation with plasma Env IgA; two are related to the indirect correlation with Env IgG responses, and three are related to cellular responses (*i.e*., peripheral blood mononuclear cell (PBMC) derived cytokines).		
1. IgA CRF01 AE. C1 peptide [12,54]	IgA binding to this C1 peptide significantly correlated with increased risk of infection among vaccinees (decreased vaccine efficacy).	Follow-up analysis of the C1 Env IgA/IgG ratio significantly correlated with decreased vaccine efficacy [54].
2. IgA A.con.env03 gp140 CF [12,54]	IgA binding to this individual Env protein correlated with increased risk of infection among vaccinees (decreased vaccine efficacy).	This measurement was part of the Plasma Env IgA Breadth Score, which was a primary variable in RV144. Follow-up analysis of the Env IgA/IgG ratio significantly correlated with decreased vaccine efficacy [54].
3. IgA A.OOMSA gp140 CF [12,54]	IgA binding to this individual Env protein correlated with increased risk of infection among vaccinees (decreased vaccine efficacy).	This measurement was part of the Plasma Env IgA Breadth Score, which was a primary variable in RV144.
**Secondary Immune Measurements** A series of immune measurements, in addition to the preselected primary six variables, were evaluated for correlations with the risk of HIV-1 infection. Of all the secondary immune variables, eight of these had a *p*-value of < 0.05. Three of these are related to the direct correlation with plasma Env IgA; two are related to the indirect correlation with Env IgG responses, and three are related to cellular responses (*i.e*., PBMC derived cytokines).		
4. IgG A244gD-293T gp120 [12]	IgG binding to vaccine strain Env correlated with a decreased vaccine efficacy.	This supports IgA/Avidity interaction model, where avidity includes binding to A244 Env. Follow-up analysis of the Env IgA/IgG ratio significantly correlated with decreased vaccine efficacy [54].
5. Composite V2 microarray (hotspot) [12,15].	IgG binding to the V2 composite score from the peptide microarray correlated with a decreased risk of infection.	This supports the V1/V2 IgG primary immune measurement that correlated with a decreased risk of infection [12], the V2 virus sieve analysis [24] and the follow-up analysis to examine linear V2 by the binding antibody multiplex assay.
6. PBMC Luminex Score [12]	The cytokine score from Env stimulated PBMC supernatants correlated with a decreased risk of HIV-1 infection.	Individual cytokines (IL-10 and IL-13), as indicated below, correlated with a decreased risk of HIV-1 infection.
7. IL-10 [12] and 8. IL-13 [12]	These individual cytokine responses from Env stimulated PBMC supernatants correlated with a decreased risk of HIV-1 infection.	Single cell transcriptomics demonstrated that RV144 vaccine-specific T-cells produced this cytokine [69].

#### 4.2.5. Multiple Antibody Specificities and Function: Vaccine Efficacy

Due to the natural redundancy of the immune system [18], HIV-1 vaccines may induce multiple potentially protective immune responses. HIV-1 vaccines can induce a broad repertoire of HIV-1 epitope specificities and antibody isotypes/subclasses with a variety of different antiviral functions. Even in RV144, where V1/V2 Env IgG responses were identified as an immune correlate of risk of HIV-1, there was broad heterogeneity among vaccines in their immune responses with a diverse repertoire of antibody forms, specificities and functions [63]. Some of these diverse immune measurements were not tested in the immune correlates analysis, due to inherent assay limitations, sample limitations and the requirement of minimizing test variables in order to maintain statistical power. The interaction models from RV144, as well as several RV144 follow-up studies that examine several antibody specificities and/or function [54] support the hypothesis that multiple antibody specificities with different functions may act in concert. Evaluating multiple antibody specificities and antiviral functions, in addition to identifying single correlates of infection risk, will be central to identifying mechanistic correlates of protection for HIV-1 toward the goal of identifying an efficacious vaccine strategy. 

## 5. Time-Dependent Immune Measurements and Vaccine Efficacy

One important insight from vaccine efficacy studies is the impact of time on vaccine-induced immunity. The time point at which vaccine efficacy is determined in a trial can dramatically influence the resulting level of vaccine efficacy. RV144 had a 60.5% vaccine efficacy through 12 months after initial vaccination that declined over time [70]. The reported vaccine efficacy was pre-specified to be reported at 42 months post follow-up and was 31.2% at that time*.* This higher level of vaccine efficacy at an earlier time point is hypothesized to be due to a higher level or quality of antibody response that decreased over time For example, Env IgG3 responses are induced in RV144, but decline more rapidly than the overall Env IgG response [14]. Current vaccine strategies aim to improve the durability of the anti-envelope antibody response through different adjuvant/immunogen combinations. Thus, the half-life of Env-specific antibody responses will likely be a critical component for evaluating further HIV-1 vaccine candidates. Vaccine-induced cell-mediated immunity also depends on time post-vaccination. Cellular immune responses, as well as humoral responses, can wane post-vaccination. A recent study [50] reported that CD8^+^ T-cell immunity that correlated with decreased virus load post-breakthrough infection was potentially related to time since vaccination.

## 6. Conclusions

HIV-1 vaccines that can induce an orchestrated response comprised of all arms of the immune response (innate, cellular and humoral) may prove to be the most efficacious across different populations, genders and routes of transmission. Thus, it is imperative to advance forward with strategies for both effective T- and B-cell responses that likely will be induced through specific engagement of the innate immune system. New technologies and approaches have allowed the field to cast a broader net in evaluating vaccine strategies. The advent of virus sequencing at the single genome level has allowed for a detailed analysis of the specificities of immune responses that can impact virus replication in both human and non-human primates. These virus-specific approaches for that are not sensitive to vaccine-induced immunity compared to those viruses that are sensitive; (2) potential insights into important vulnerable sites (and their structure) on the virion that vaccines should target; and (3) information on whether the described vaccine-induced humoral and cellular immune responses have epitope specificities that match those identified as sieve sites in the virus. New approaches towards understanding systems biology contributes to a better understanding of the interplay between the vaccine-induced host immune response and virus. Taken together, these technological advances have yielded a robust amount of data that has an unprecedented need for innovation in the way the data are analyzed.

Analysis of host genetics, viral genetics and immune responses [71,72] have already provided numerous insights into both innate and adaptive responses of virus control and disease progression in HIV-1 infected individuals. Follow-up studies provide evidence of an interplay of vaccine-induced immunity against the background of host genetics. Understanding the role of host genetics and the functional attributes of vaccine-induced immunity can enable a better understanding of which immune correlates will be broadly applicable in a globally efficacious vaccine. Similarly, studies are ongoing to understand if immune responses to one vaccine can be predicted by immunity to another vaccine; thus, also allowing deeper insights into intrinsic population effects that may not be distinct to the vaccine strategy being tested.

Currently licensed vaccines for other pathogens are based on empirical studies, and many do not have defined mechanistic correlates of protection. The protective efficacy of some of these vaccines are associated with antibody responses, with the exception of rotavirus and BCG ((Bacillus Calmette-Guerin) tuberculosis vaccine) [18,73]. Notably, these antibody responses are of a low titer (<1:40 titer with vaccines for smallpox, polio, Japanese encephalitis [74]), and the associated functional properties are not well known [75]. Mucosal IgG has been associated with the protection of several vaccines (e.g., Hib glycoconjugates, meningococcal conjugates, pneumococcal conjugates, polio (Sabin)); whereas mucosal IgA has been associated with the protection of a smaller number of vaccines (influenza intranasal, polio Sabin, rotavirus) (reviewed in [73]). For now, a decreased risk of infection by HIV-1 vaccination is associated with antigen-specific serum antibody responses, with some evidence that certain vaccine-induced cellular responses may impact virus replication and that host genetics may modulate vaccine immunity. Analyses of the correlates of HIV-1 infection risk have demonstrated that there are intersections among different analyses (involving immune responses, virus and host genetics), thus providing the areas of highest information content for understanding the potential mechanisms of protective immunity. Identification and rigorous characterization of these areas of overlap are likely to be transformative for the next phase of HIV-1 vaccine design and evaluation.
